# Prevalence of Reduced Vision among UK Elderly Drivers: The Bridlington Eye Assessment Project (BEAP)—A Cross-Sectional Study

**DOI:** 10.1155/2022/8321948

**Published:** 2022-09-15

**Authors:** Craig Wilde, Ali Poostchi, Georgios D. Panos, Jonathan G. Hillman, Hamish K. MacNab, Harminder Dua, Winfried M. Amoaku, Stephen A. Vernon

**Affiliations:** ^1^Department of Ophthalmology and Vision Sciences, Division of Clinical Neurosciences, B Floor, EENT Centre, Queen's Medical Centre, Nottingham University Hospitals and University of Nottingham, Nottingham, UK; ^2^The Medical Centre, Station Avenue, Bridlington YO16 4LZ, UK

## Abstract

Self-assessment of driving fitness is mandatory in the United Kingdom. A paucity of data on visual function among drivers exists. We report prevalence of elderly drivers below legal visual acuity (VA) standard from a population study (The Bridlington Eye Assessment Project (BEAP)) conducted from 2002 to 2006. All residents aged ≥65 years were invited, 3459 undergoing structured interviews/ophthalmic examinations. Driving status was recorded, VA measured, and visual field (VF) testing performed. Outcomes were prevalence and characteristics of drivers below VA legal standard and prevalence of bilateral VF defects. Conditions causing reduced VA were explored and those with treatable conditions allowing visual improvement identified. Duration since last optometry review was recorded. Associations were explored using unpaired *t*-tests for continuous and chi-squared for discrete variables. Logistic regression was used for multivariate analysis and to determine odd ratios in the final adjusted model. Statistical analysis was performed using Stata 14.0 (Stata Corp, Tx). Within this sample, 7.1% (95% CI 6.0–8.3) of drivers fell below the VA legal driving standard (6/12) in their better eye, with 20% not having seen an optometrist for 2 years, including 8.2% who had not attended for over 5 years. The percentage of drivers falling below the VA minimum increases with age reaching 22.8% (95% CI 13.7–35.3) among those aged 85–89 years. 7.2% (95% CI 6.2–8.6) of drivers had bilateral visual field defects. 93% of drivers with reduced VA below legal standard had a cataract, refractive error or both in at least one eye. Significant numbers of elderly drive with VA below legal standard, most having easily correctable causes. Poor attendance with optometrists appears commonplace. Public education raised awareness of legal driving standards and encouraged compliance are required. Regular eye tests, appropriate refractive correction, and cataract surgery when needed should be encouraged.

## 1. Introduction

With the aging Western population, older adults form the fastest growing demographic with a trend to drive in later life [1]. Motor vehicle collisions (MVC) involving the elderly appear more likely to be severe or involve fatalities [2]. Although driving is complex, requiring cognitive and physical inputs, it inarguably requires adequate visual function, but the level at which driving becomes dangerous has been debated, as is the best method of risk assessment. Several types of visual impairment increase risk of MVC [3]. Reduced contrast sensitivity in older adults with cataract is consistently associated with increased accident rates and reduced driving performance [4, 5], along with visual field (VF) defects [6, 7] and slow visual processing speed [8–11]. Reduced visual acuity (VA) has a weaker relationship to driving safety [6, 12]. Some older drivers voluntarily stop because of visual problems, either of their own volition or following advice or encouragement from friends, relatives, or health professionals [13–15]. Some modify driving behavior, placing self-imposed restrictions to reduce risk, including reducing mileage, terminating night driving, driving in unfamiliar locations or bad weather, long journeys, or fast roads [13, 16–21]. Others give up when their licenses are not renewed if they fail to demonstrate compliance with minimum visual requirements of the licensing authority.

In the United Kingdom (UK), European Union (EU) and most countries, legal minimum driving visual standards exist. In the United Kingdom, compliance is demonstrated at the start of the driving test, by reading a car number plate (made after September 01, 2001) from 20 meters, with correction from distance glasses or contact lenses if required. After this, no further check is performed by the Driver and Vehicle Licensing Agency (DVLA). Driving license renewal is every 10 years until the age of 70, after which it is every 3 years. In the process of renewal and in-between, the DVLA relies on self-assessment of fitness to drive and self-reporting failure to meet visual standards. To the best of our knowledge, no data exist from population-based studies evaluating how many road users currently drive with VA below the UK minimum.

We report the results of a UK-based population study, estimating the proportion of licensed drivers aged ≥65 years who fail to meet the current VA standard and the number who continue driving despite possible awareness of reduced vision. We discuss ophthalmological conditions associated with driving with VA below the legal requirement, and whether these are correctable. We report factors that influence whether study participants will be a current driver.

## 2. Materials and Methods

### 2.1. Study Population

The Bridlington Eye Assessment Project (BEAP) study methodology is described in detail elsewhere [22]. Briefly, BEAP is a population-based study to determine prevalence of common eye diseases within the elderly population using clinical examination by optometrists, visual field testing, and digital imaging technology. Primary ophthalmic diseases studied were age-related macular degeneration (AMD), cataract, and glaucoma.

Briefly, all individuals aged ≥65 years and registered with general practice surgeries within the town of Bridlington were systematically invited to attend a screening visit. Individuals known to be registered blind (severely visually impaired [SVI]) or partially sighted (visually impaired [VI]), bed-bound or with significant dementia and those known to be moving in or out of the area during the study were excluded. Participants were invited by letter on a street-by-street basis (in ascending numerical order of postcode) to telephone the project and make an appointment to be examined. Study recruitment occurred between November 05, 2002 and March 29, 2006. All participants were interviewed, in person, by a trained research nurse using a structured questionnaire, and examined by one of four specially trained optometrists with structured proforma completed by the research staff. In total, 3549 individuals participated in the initial study examination, corresponding to 56% of the eligible study population.

### 2.2. Questionnaire

All participants were interviewed by a research nurse, demographic data obtained and a structured questionnaire covering a history of diabetes, stroke, and hypertension, together with drug history performed. Any history of amblyopia, ocular surgery, or other eye disease was recorded. Spectacle requirements were documented. All participants were specifically asked if they were current drivers. Only participants who were present-day motorists were recorded as drivers. Individuals who had previously driven but given up were recorded as nondrivers. Social history, including number of household co-inhabitants, was also recorded. Participants were asked if they were satisfied with their current level of vision and questioned when they last visited their optometrist for routine ophthalmological examination.

### 2.3. Ocular Examination

For each eye, VA LogMAR measurements were obtained (Bailie Lovie number 4 chart) uncorrected, with distance glasses and pinhole. Habitual VA was recorded using current spectacles or contact lenses, representing daily driving vision. Habitual VA for the better eye was used in analyses. Acuity and visual field testing were performed by the research nurse. If the eye with better acuity failed to achieve LogMAR 0.3 (Snellen 6/12) with or without spectacle correction, it was deemed this would induce a failure to pass the DVLA acuity standard.

Full eye examination including slit lamp and mydriatic fundal assessment was performed by one of four specially trained optometrists who were unaware of the health questionnaire results, including driving status. Participants were dilated and the Lens Opacities Classification 3 system (LOCS 3) used to grade cataracts [23]. The optic disc, macula, and peripheral retina were examined using slit lamp biomicroscopy and 90D lens.

### 2.4. Visual Fields

VF tests were performed in each eye during the initial visit using a Henson Pro 5000 automated perimeter, software v3.1.4 (Tinsley Instruments, Croydon, UK). A single-stimulus, suprathreshold, central 26-point strategy was employed. The test was extended to 68-points if defects were detected. The perimeter automatically graded outputs as normal, suspect, or defect. Any defect was treated as abnormal with onward referral for hospital assessment and full-threshold Henson test. Results from screening visits and hospital follow-ups, including VF printouts and fundus images were reviewed and classified by three ophthalmologists including a trained glaucoma subspecialist (SAV), who acted as final arbiter. Where a defect disappeared on repeat testing or where no clear cause was found, it was classified as artifactual. In all other cases, the cause was identified and recorded. The presence of bilateral screening defects together with confirmed defects at clinic follow-up was deemed possibly significant for driving.

Those with new or unexplained ocular pathology including raised intraocular pressure, suspicious/abnormal VF, or reduced vision were referred to the hospital eye service for assessment and outcomes recorded at each visit in a prospective longitudinal manner until definitive diagnosis was established. The time frame was determined on a case by case basis and the underlying pathology.

The study received ethics committee approval (Scarborough and North-East Yorkshire Local Ethics Research Committee; Ref No. PB/RH/02/288). Its methodology adhered to the tenets of the Declaration of Helsinki. Informed consent was obtained from all participants.

### 2.5. Data Analysis

Associations between groups (drivers and nondrivers) were explored using unpaired *t*-tests for continuous, and chi-squared for discrete variables. Where necessary, results were stratified using Mantal Haenszel methods. Logistic regression was used for multivariate analysis and to determine odd ratios in the final adjusted model. To explore the influence of interaction and effect modification between variables, we used a likelihood ratio test to compare adjusted model nested with and without the interaction term. We performed complete case analysis in the multivariate models. Confidence intervals for proportions of drivers were calculated using the modified Wald method. All analysis was performed using Stata v14 (Stata Corp, Tx).

## 3. Results

In total, 3549 individuals participated in the study examination, corresponding to 56% of the eligible population. Basic demographic information was available for all participants within the sampling frame. Gender balance was similar for attenders and nonattenders with a small difference in average Jarman score (−3.92 vs −1.75) corresponding to slightly higher levels of deprivation among nonattenders. This was not judged to be clinically significant. At recruitment, nonattenders were slightly older, (mean: 75.3 vs 73.4 years) a difference common among population-based studies, attributed to older individuals being reluctant to attend assessments. Driving status, VA or VF, was absent for 35 participants who were excluded from analysis. Reasons for missing data included nonparticipation and/or failure to complete VF and VA testing and failure of data entry. It was assumed data were missing at random, with low risk of subsequent bias.

The study population age distribution was skewed toward the lower end, with 2743 (78%) participants being in their 60s or 70s. Only 34 (<1%) attenders were 90 years or older (Table 1). Similarly, 1629 (85%) drivers were in their 60s and 70s, and only 15% of drivers were aged ≥80 years. Only 2 individuals (out of a total of 1919 drivers, 0.1%) aged 90 years or over continued to drive. The population had an overall female preponderance (56% vs 44%), but driving prevalence was lower among women (36%) than men (79%).

Driving prevalence declined with age (adjusted odds ratio [OR] per year increase 0.93, 95% confidence interval [CI] 0.92–0.94, *p* < 0.001). Within the 65 years or over population gender had the greatest impact on predicting whether an individual would drive, with being male associated with increased likelihood (OR 7.18, 95% CI 6.07–8.49, *p* < 0.001). Elderly participants living alone were more than twice as likely not to drive, with a crude OR of 0.43 (95% CI 0.37–0.49, *p* < 0.001). With adjustment for age this effect largely disappeared (adjusted OR 0.84, 95% CI 0.71–1.0, *p*=0.05), reflecting age as an important confounder. Participants who were happy with their vision were more likely to drive (adjusted OR 1.46, 95% CI 1.21–1.75, *p* < 0.001). When adjusted for age, participants whose VA was ≥6/12, with no bilateral VF defect were more likely to drive (OR 1.95, 95% CI 1.58–2.41, *p* < 0.001). General health problems influenced driving behavior, with a history of stroke reducing the likelihood of being a current driver (adjusted OR 0.66, 95% CI 0.50–0.86, *p*=0.003). Hypertension (adjusted OR 0.9, 95% CI 0.74–1.1, *p*=0.06) or a history of diabetes mellitus (DM) did not influence the decision to drive (OR 1.06). Similarly, whether a subject had undergone cataract surgery in either eye (after adjustment for age) did not influence the decision to stop driving (OR 0.94, 95% CI 0.7–1.2).

In this population, 7.1% (95% CI 6.0–8.3) of drivers did not reach the VA threshold (6/12) for legal driving standards in their better eye, either unaided or with habitual spectacle correction. Most were within 1 Snellen line of the required vision, but approximately 8% had a VA that was worse than legal standard by 2 lines or more (Table 2). With pinhole correction, only 2.5% (95% CI 1.8–3.3) of drivers failed to reach this threshold.

Of the 1595 nondrivers within the population, a higher proportion had habitual VA of LogMAR 0.3 (Snellen 6/12) or worse (18.7%). However, with logistic regression, when controlled for age, being a nondriver was not associated with being more likely to have habitual VA worse than LogMAR 0.3 (6/12) (OR 1.02, 95%CI-0.96-1.08). Overall, based on habitual VA, data from this population suggest the yield of drivers not meeting the VA criteria would be 7%, 8.3%, 11.2%, 14.5%, and 23.7% for the 65+, 70+, 75+, 80+, and 85+ age groups, respectively.

Of the 136 drivers whose VA fell below the current legal standard, 79 (58%) were male, while 57 (42%) were female. When adjusted for age, gender did not influence the likelihood of driving with reduced habitual VA (OR 0.89, 95%CI 0.42–1.94). With increasing age, the percentage of drivers who fail to meet the VA minimum increases significantly, with 22.8% (95% CI 13.7–35.3) of drivers in the 85-89-year category falling below the legal VA minimum. Out of the 2 road users over 90 years, 1 (50%) failed to meet the legal standard (Table 2).

Bilateral VF defects were present in 240 of 1919 (12.5%) drivers at the initial visit, with 54 of these participants (22.5%) being normal on re-test. Only 7.2% (95% CI 6.2–8.6) of the driving population had confirmed bilateral defects at subsequent hospital visits. Confirmed bilateral VF defects and/or reduced acuity with habitual correction affected 12.5% of drivers, while confirmed defects and/or reduced acuity with pinhole affected 8.2% of drivers.

Living alone appeared to have an important effect on the decision to drive. Those living by oneself were older than those who lived with others, and social isolation appeared to increase the chance of driving with vision below legal minimum driving standard (17%) compared to those who lived with others (10%). This effect modification appeared to negate the influence of impaired vision on the decision to drive and there was a significant difference (*p*=0.002) between adjusted models with and without the interaction term. The effect was similar for men and women.

Of the 136 individuals who continued to drive with VA below the minimum required standard, 50 participants (36.8%) reported being dissatisfied with their vision. Twenty (20) percent of drivers with VA below the legal standard had not seen an optometrist for 2 or more years, including 8.15% who had not been for over 5 years.

Seventy-six (76) of the 136 (55.9%; 95% CI 47.5–64.0) ineligible drivers had reduced VA in at least one eye secondary to cataract. Fifty (36.8%; 95% CI 29.1–45.1) had a refractive error in at least one eye, 6 (4.4%; 95% CI 1.8–9.5) had posterior capsular opacification, 2 participants had AMD as the cause of reduced VA in their better eye, and another had corneal scarring. In this population, 93% of ineligible drivers had reduced VA in at least one eye from either a cataract or refractive error, or combination of the two.

## 4. Discussion

To the best of our knowledge, this report is the first large population study to examine the proportion of elderly drivers within a UK population who currently drive with reduced VA below legal minimum standards, offering invaluable information, despite the limitations discussed below, that until now remained unavailable.

Gender plays a significant role on driving status among the elderly, with a male preponderance of road users within the over 65-year population. This disparity likely reflects historical, cultural, and occupational characteristics, and this may evolve over time, given significant shifts in family and occupational dynamics. Males may have been more likely to have learned to drive and drive for vocational purposes. The observed differences may reflect this and no inference can be made regarding the influence of gender on driving cessation within this population. Previous studies, however, suggest males continue to drive for greater distances and for longer [24] with females more likely to give up earlier [24]. Males have been reported as greater risk takers, which may explain their increased driving longevity [24].

This study indicates a notable part of the elderly population drives with habitual VA below the legal minimum standard. The proportion of current drivers with VA blow legal minimum standard increases with age. Our findings are comparable to previous contemporaneous population studies from America and Australia that report reduced VA of a similar magnitude [25, 26]. Within the wider BEAP population, levels of VA impairment are similar between driving and nondriving cohorts when controlled for age. If self-regulation to current VA driving standards was adhered to, or was effective, one would expect prevalence of VA impairment to be higher among nondrivers. However, this is not the case suggesting VA has little influence on the decision to terminate driving, that individuals could be unaware of visual deficits, or other factors primarily influence decisions to stop driving. The proportion of individuals who intentionally continue to drive, despite awareness of failure to comply with legal standards is difficult to ascertain. However, a significant proportion (36.8%) of drivers with VA worse than 6/12 reported dissatisfaction with vision, demonstrating potential insight into their reduced vision to some extent. Reasons why these individuals have not had their vision evaluated or sought prior treatment are beyond the scope of this publication. We similarly cannot speculate on how many current drivers have full awareness of the legal visual driving standards as this was not assessed.

Interestingly, the number of road users with VA below legal driving standard is reduced by 65% with the use of a pinhole, indicating for the majority, the issue may be simple to overcome, with refraction or onward referral for cataract surgery. This is not surprising given low uptake of regular optometrist appointments [27], supported by our finding that 8% of elderly drivers with VA below legal minimum driving standard have not attended an optometrist for over 5 years.

Regulation by self-assessment is clearly an ineffective policy to enforce visual driving standards, being inefficient at detecting individuals falling below the current minimum VA standard. Significant numbers of elderly drivers are on the roads with vision that is suboptimal, reflecting either lack of awareness of the current visual standards or purposeful noncompliance with them. The legal driving standard should be advertised more widely at routine optometry appointments and publicly visible reminders to motorists of their obligations for self-assessment considered. The elderly should be encouraged to avail themselves of their entitlement to a regular government funded sight test at least every 2 years. Public education on driving visual standards should be pursued, for example, with use of “number plate” posters with corresponding 20m markers being accessible in public buildings, including leisure centers, libraries, or GP reception areas. A proactive attitude toward self-screening for reduced vision within the elderly driving community and campaigns encouraging elderly drivers to attend optometrists should be fostered.

Poor attendance of optometry appointments among the elderly may reflect fear of failure to meet legal driving standards, with subsequent license implications. The public should be reassured; however, as there is a high likelihood that the VA standard will be achievable either with new spectacles or cataract surgery. To encourage purposeful self-reporting, quick access cataract surgery pathways within the NHS could be established and advertised, allowing road users with poor vision a quicker return to the road.

The primary aim of this study was not to assess compliance with UK visual driving standards; hence, limitations to its methodology exist. First, no binocular VA was recorded. We acknowledge the current legal driving standard is to achieve a 6/12 level of VA using both eyes. Previous research, based on small samples of young adults with equal VA in both eyes, demonstrates a binocular advantage of 10%–12% under high luminance, high contrast conditions [28, 29]. However, a large-scale study of over 2500 participants concluded binocular VA can closely be predicted by monocular acuity of the better eye [30]. Among participants with less than one-line VA difference between the two eyes, average binocular summation was a gain of 0.03 logMAR units or 1.5 letters. For individuals with more unequal VA between eyes, on average, less than one letter difference between monocular and binocular acuities existed, with equal numbers demonstrating binocular inhibition and summation [30]. The authors report that although binocular advantage is statistically significant, it is about half that reported previously and of unlikely clinical significance [30]. Our data may potentially overestimate ineligible road users in reference to the VA criteria alone. However, this will likely be negligible.

Another limitation is that, in addition to the Snellen 6/12 VA minimum, drivers must be able to read a number plate (post September 2001 format, comprising 79 mm by 50 mm letters) at 20m in good light with both eyes. In our study, this was not assessed. We acknowledge that because of differing conditions of contrast and glare, VA as assessed on a chart in a clinic room does not correspond completely to being able to read a number plate. Those who demonstrate a VA of 6/7.5 Snellen or +0.10 logMAR or better will likely pass the number plate test [31]. One study suggested an overlap zone, where individuals with VA between 6/9 to 6/12 Snellen or +0.30 to +0.2 logMAR may not have agreement between the two methods. And 2% could meet the VA requirement but subsequently fail the number plate test [31]. Similar participants in our study will remain undetected, but the number will be small. We note at the time of this study, driving VA standard was defined solely by compliance with the number plate test. It was not until 2012 that the VA standard of at least 6/12 was added. For this study, we used the more stringent post 2012 criteria, further underestimating the true number of noncompliant drivers. This was a pragmatic decision in part reflecting that as a secondary analysis, the primary aim of which was not a study of driving, formal binocular assessment of the ability to read number plates was not undertaken.

With reliance on self-reported driving status results could be subject to reporting bias, with drivers aware of reduced VA falsely reporting they do not currently drive, recognizing this as the socially desirable and legally compliant answer. This is unlikely, however, as the study aim was not related to driving. We acknowledge lack of information on driving status and VA of nonparticipants and these may have different parameters. However, they have similar sociodemographic and gender backgrounds.

Current UK DVLA standards for group 1 drivers demands VF extends 120° horizontally, with 50° either side fixation, with no significant defect within 20° from fixation, but this is not routinely assessed. Unlike home testing with a number plate, drivers cannot self-perform VF tests, relying on the assumption that in the absence of bilateral eye disease they are likely compliant. Assessment is by means of the Esterman program, often at the request of the DVLA following driver declaration of pathology that may affect VF, including glaucoma, post panretinal laser photocoagulation, or stroke. A number of participants had bilateral VF defects, but these were identified during monocular testing. It is likely some of these individuals would fail the Esterman test and number of ineligible drivers within the Bridlington population would increase. Failure to identify a precise number of these individuals within our study is a further limitation.

Although this study found a minority of elderly drivers had VA below legal standard, comparisons of MVC data from the Bridlington area show rates are no greater for older road users when compared to younger age groups. We used the Office of National Statistics, to compare road safety data from 2005 for the East Riding of Yorkshire, from where our sample was drawn, to data for all local authority regions. For those 65 years and older the standardized accident rate was 0.20% in East Riding and 0.21% nationally. For those aged 15–64 years, the standardized accident rates were 0.71% in East Riding and 0.78% nationally [32]. Differences may reflect a myriad of factors, being visual or otherwise. Explanations may include driving modifications among the elderly, such as not driving in darkness or reduced miles driven. No visual acuity data are available for drivers under 65 years in our population and therefore inferences and comparisons regarding the influence of vision on accident rates cannot be made within this study. Furthermore, we cannot establish whether older cohorts have more crashes per miles driven. Previous literature suggests drivers with VA between 6/12 and 6/30 are not at elevated risk of MVC [7]. The authors encourage a VA minimum but emphasize the 6/12 cut-off is arbitrary, with little evidence behind predicting risk of MVC.

Other limitations include potential for response bias. Driving habits of nonattenders may differ, particularly given their slightly older age. Our attendance rates are lower than in some studies [33–35], reflecting the older age of inclusion in our sample, but are largely comparable to those in others [36, 37]. Our large study size, adequate response rate and similarity in sociodemographic backgrounds, and gender between attenders and nonattenders are all strengths. Data from this study form part of a secondary analysis and as such do not address crash risk or driving performance. The driving parameters of younger individuals or non-Caucasian drivers remain unknown as these were not included. Lack of data on participants driving record, including miles per annum and MVC history prevents any analysis or inference on risk of driving with reduced VA, VF defects or influence of age, and other co-morbidities. This restricts our analysis to compliance with legal VA driving standards. Another limitation is that the data set is now from the previous decade. The delay in reporting our results is due to the fact that in 2012 the DVLA changed their requirements for the legal driving standard with the introduction of the Snellen VA criteria, in addition to the longer standing requirement of reading a number plate at 20 meters. This allowed a fresh review of the historical data to report a measure of how many drivers are below the current legal standard. Driving habits may have changed significantly since our study was conducted. We expect this to be particularly true for the influence of gender. However, this data remain to the best of our knowledge, the largest population-based measure of compliance among UK elderly drivers to the legal VA minimum standard. As such, we feel the use of this old data, with the associated question of whether it remains representative of current elderly drivers, still offers valuable information. Since the BEAP study was conducted no significant shift in monitoring for compliance with legal driving standards have occurred. Neither has there been significant changes in the way ophthalmology and optometry care is delivered within the United Kingdom, with no major modification preventing access to health care. When this study was conducted no artificial restrictions prevented cataract surgery on the basis of acuity, as was occurring in 57% of Primary Care Trusts in England in 2013 [38]. Rationing surgery by acuity continues in some regions in 2019 despite National Institute for Health and Care Excellence (NICE) guidance in 2017 suggesting this should not occur [39]. With demand for cataract surgery in the United Kingdom predicted to rise by 50% in the next 20 years [40], it is vital that improved access to surgery occurs.

## 5. Conclusions

In conclusion, although further study on driving status of the elderly is required, including studies involving performance on driving simulators, as has been used in patients with glaucoma and neurological field defects [41], this study suggests significant numbers of older drivers have VA below the legal requirement, and the likelihood of driving illegally increases with age. Those living alone are at risk since they may lack options or support to facilitate driving cessation. Increased public awareness of legal driving standards is required, with drivers encouraged to attend optometrists regularly for appropriate refractive correction. Prompt referral for cataract surgery in suitable persons will enhance their vision, and help meet legal driving requirements.

## Figures and Tables

**Table 1:** Age- and gender-specific prevalence of driving.

		Age group (years)						Total
		65–69	70–74	75–79	80–84	85–89	≥90	
Male drivers	Yes	329 **(85.7; 81.5-88.9)**	430 **(85.8; 82.5-88.6)**	264 **(77.4; 72.7-81.6)**	151 **(65.1; 58.5-70.9)**	43 **(58.9; 47.4-69.5)**	2 **(16.7; 3.5-46.0)**	1219 **(79; 76.9-81.0)**
	No	55 (14.3)	71 (14.2)	77 (22.6)	81 (34.9)	30 (41.1)	10 (83.3)	324 (21.0)
	Total	384	501	341	232	73	12	1543 (43.91)

Female drivers	Yes	221 **(47.8, 43.3-52.4)**	245 **(42.8; 38.8-46.8)**	140 **(29.1; 25.2-33.3)**	80 **(25.6; 21.0-30.7)**	14 **(11.6; 7.0-18.9)**	0 **(0.0; 0.0-17.6)**	700 **(35.5; 33.4-37.7)**
	No	241 (52.16)	328 (57.24)	342 (70.95)	233 (74.44)	105 (88.24)	22 (100)	1271 (64.49)
	Total	462	573	482	313	119	22	1971 (56.09)

Total number of drivers within each age-range		550 **(65.0; 61.7-68.2)**	675 **(62.9; 59.9-65.7)**	404 **(49.1; 45.7-52.5)**	231 **(42.4; 38.3-46.6)**	57 **(29.7; 23.7-36.5)**	2 **(5.9; 0.65-20.1)**	1919 **(54.6; 53.0-56.3)**

Combined total of participants-drivers and nondrivers		846 (24.1)	1074 (30.6)	823 (23.4)	545 (15.5)	192 (5.5)	34 (1.0)	3514

Data are number (percentage; 95% confidence interval).

**Table 2:** Variation of VA level among all drivers who fall below the DVLA VA minimum requirement, stratified for age.

Age group (years)	Stratified habitual visual acuity (logMAR)					Total number of ineligible drives	Total number of ineligible with pinhole VA	Total
	+0.3	+0.4	+0.5	+0.6	+0.8			
65–69	10	9	2	1	0	22 **(4.0; 2.6–6.0)**	5 (0.9; 0.32–2.2)	550
70–74	16	8	8	4	0	36 **(5.3; 3.0–7.1)**	13 (1.9; 1.1–3.3)	675
75–79	13	10	9	4	0	36 **(8.9; 6.5–12.1)**	13 (3.2; 1.8–5.5)	404
80–84	10	15	3	0	0	28 **(12.1; 8.5–17.0)**	9 (3.9; 2.0–7.3)	231
85–89	5	4	2	1	1	13 **(22.8; 13.7–35.3)**	7 (12.3; 5.8–23.6)	57
≥90	1	0	0	0	0	1 **(50.0; 8.5–91)**	0 (0; 0–71)	2
Total	55 **(40.4; 32.6–48.9)**	46 **(33.8; 26.4–42.1)**	24 **(17.6; 12.1–25.0)**	10 **(7.4; 3.9–13.2)**	1 **(0.74; ˂0.01–4.6-)**	**136 (7.1; 6.0–8.3)**	47 **(2.5; 1.8–3.3)**	**1919**

Data are number (percentage; 95% confidence interval).

## Data Availability

Data are available from the authors upon request.

## References

[1] Foley D. J., Heimovitz H. K., Guralnik J. M., Brock D. B. (2002). Driving life expectancy of persons aged 70 years and older in the United States. *American Journal of Public Health*.

[2] Evans L. (1988). Risk of fatality from physical trauma versus sex and age. *The Journal of Trauma*.

[3] Owsley C., McGwin G. (2010). Vision and driving. *Vision Research*.

[4] Owsley C., Stalvey B. T., Wells J., Sloane M. E., McGwin G. (2001). Visual risk factors for crash involvement in older drivers with cataract. *Archives of Ophthalmology*.

[5] Wood J. M., Carberry T. P. (2006). Bilateral cataract surgery and driving performance. *British Journal of Ophthalmology*.

[6] Johnson C. A., Keltner J. L. (1983). Incidence of visual field loss in 20, 000 eyes and its relationship to driving performance. *Archives of Ophthalmology*.

[7] McGwin G., Xie A., Mays A. (2005). Visual field defects and the risk of motor vehicle collisions among patients with glaucoma. *Investigative Ophthalmology & Visual Science*.

[8] Ball K. K., Roenker D. L., Wadley V. G. (2006). Can high-risk older drivers be identified through performance-based measures in a department of motor vehicles setting?. *Journal of the American Geriatrics Society*.

[9] Owsley C., Ball K., McGwin G. (1998). Visual processing impairment and risk of motor vehicle crash among older adults. *JAMA*.

[10] Rubin G. S., Ng E. S. W., Bandeen-Roche K., Keyl P. M., Freeman E. E., West S. K. (2007). A prospective, population-based study of the role of visual impairment in motor vehicle crashes among older drivers: the SEE study. *Investigative Ophthalmology & Visual Science*.

[11] Ball K., Owsley C., Sloane M. E., Roenker D. L., Bruni J. R. (1993). Visual attention problems as a predictor of vehicle crashes in older drivers. *Investigative Ophthalmology & Visual Science*.

[12] Keeffe J. E., Jin C. F., Weih L. M., McCarty C. A., Taylor H. R. (2002). Vision impairment and older drivers: who’s driving?. *British Journal of Ophthalmology*.

[13] Keay L., Munoz B., Turano K. A. (2009). Visual and cognitive deficits predict stopping or restricting driving: the salisbury eye evaluation driving study (SEEDS). *Investigative Ophthalmology & Visual Science*.

[14] Ragland D. R., Satariano W. A., MacLeod K. E. (2004). Reasons given by older people for limitation or avoidance of driving. *The Gerontologist*.

[15] Campbell M. K., Bush T. L., Hale W. E. (1993). Medical conditions associated with driving cessation in community-dwelling, ambulatory elders. *Journal of Gerontology*.

[16] Stutts J. C. (1998). Do older drivers with visual and cognitive impairments drive less?. *Journal of the American Geriatrics Society*.

[17] Ball K., Owsley C., Stalvey B., Roenker D. L., Sloane M. E., Graves M. (1998). Driving avoidance and functional impairment in older drivers. *Accident Analysis & Prevention*.

[18] McGwin G., Chapman V., Owsley C. (2000). Visual risk factors for driving difficulty among older drivers. *Accident Analysis & Prevention*.

[19] Freeman E. E., Muñoz B., Turano K. A., West S. K. (2006). Measures of visual function and their association with driving modification in older adults. *Investigative Opthalmology & Visual Science*.

[20] Lyman J. M., McGwin G., Sims R. V. (2001). Factors related to driving difficulty and habits in older drivers. *Accident Analysis & Prevention*.

[21] Kaleem M. A., Munoz B. E., Munro C. A., Gower E. W., West S. K. (2012). Visual characteristics of elderly night drivers in the salisbury eye evaluation driving study. *Investigative Opthalmology & Visual Science*.

[22] Wilde C., Poostchi A., Mehta R. L. (2017). Prevalence of age-related macular degeneration in an elderly UK caucasian population-the Bridlington eye assessment project: a cross-sectional study. *Eye*.

[23] Chylack L. T., Wolfe J. K., Singer D. M. (1993). The lens opacities classification system III. *Archives of Ophthalmology*.

[24] Brabyn J. A., Schneck M. E., Lott L. A., Haegerstrom-Portnoy G. (2005). Night driving self-restriction: vision function and gender differences. *Optometry and Vision Science*.

[25] Klein R., Klein B. E., Linton K. L., De Mets D. L. (1991). The beaver dam eye study: visual acuity. *Ophthalmology*.

[26] Chia E. M., Mitchell P., Rochtchina E., Foran S., Wang J. J. (2003). Unilateral visual impairment and health related quality of life: the blue mountains eye study. *British Journal of Ophthalmology*.

[27] Shickle D., Farragher T. M. (2015). Geographical inequalities in uptake of NHS-funded eye examinations: small area analysis of Leeds, UK. *Journal of Public Health*.

[28] Cagenello R., Halpern D. L., Arditi A. (1993). Binocular enhancement of visual acuity. *Journal of the Optical Society of America*.

[29] Home R. (1978). Binocular summation: a study of contrast sensitivity, visual acuity and recognition. *Vision Research*.

[30] Rubin G. S., Munoz B., Bandeen-Roche K., West S. K. (2000). Monocular versus binocular visual acuity as measures of vision impairment and predictors of visual disability. *Investigative Ophthalmology & Visual Science*.

[31] Latham K., Katsou M. F., Rae S. (2015). Advising patients on visual fitness to drive: implications of revised DVLA regulations. *British Journal of Ophthalmology*.

[32] (2018). https://www.gov.uk/government/collections/road-accidents-and-safety-statistics.

[33] Mitchell P., Smith W., Attebo K., Wang J. J. (1995). Prevalence of age-related maculopathy in Australia. *Ophthalmology*.

[34] Klein R., Klein B. E., Linton K. L. (1992). Prevalence of age-related maculopathy. *Ophthalmology*.

[35] Jonasson F., Arnarsson A., Sasaki H., Peto T., Sasaki K., Bird A. C. (2003). The prevalence of age-related maculopathy in Iceland: reykjavik eye study. *Archives of Ophthalmology*.

[36] Augood C. A., Vingerling J. R., de Jong P. T. (2006). Prevalence of age-related maculopathy in older Europeans: the European eye study (EUREYE). *Archives of Ophthalmology*.

[37] Akuffo K. O., Nolan J., Stack J. (2015). Prevalence of age-related macular degeneration in the Republic of Ireland. *British Journal of Ophthalmology*.

[38] Adams S. (2013). NHS admits widespread restrictions on cataract surgery: the telegraph. https://www.telegraph.co.uk/news/health/news/9803677/NHS-admits-widespread-restrictions-on-cataract-surgery.html.

[39] National Institute for Health and Care Excellence (NICE) (2017). Cataracts in adults: management: NICE guideline [NG77]. https://www.nice.org.uk/guidance/ng77.

[40] The Royal College of Ophthalmologists [Internet] (2019). The way forward: options to help meet demand for the current and future care of patients with eye disease. http://www.rcophth.ac.uk/wp-content/uploads/2015/10/RCOphth-The-Way-Forward-Cataract-300117.pdf.

[41] Ungewiss J., Kübler T., Sippel K. (2018). Agreement of driving simulator and on-road driving performance in patients with binocular visual field loss. *Graefes Archive for Clinical and Experimental Ophthalmology*.

